# Longitudinal Analysis of Amyloid PET and Brain MRI for Predicting Conversion from Mild Cognitive Impairment to Alzheimer’s Disease: Findings from the ADNI Cohort

**DOI:** 10.3390/tomography11030037

**Published:** 2025-03-19

**Authors:** Do-Hoon Kim

**Affiliations:** Department of Nuclear Medicine, Daejeon Eulji Medical Center, Eulji University School of Medicine, Daejeon 35233, Republic of Korea; k8016851@eulji.ac.kr; Tel.: +82-42-611-3591

**Keywords:** imaging biomarkers, Alzheimer’s disease, shape feature, Alzheimer’s disease neuroimaging initiative cohort

## Abstract

Background/Objectives: This study aimed to investigate the predictive power of integrated longitudinal amyloid positron emission tomography (PET) and brain magnetic resonance imaging (MRI) data for determining the likelihood of conversion to Alzheimer’s disease (AD) in patients with mild cognitive impairment (MCI). Methods: We included 180 patients with MCI from the Alzheimer’s Disease Neuroimaging Initiative, with baseline and 2-year follow-up scans obtained using F-18 florbetapir PET and MRI. Patients were categorized as converters (progressing to AD) or nonconverters based on a 6-year follow-up. Quantitative analyses included the calculation of amyloid burden using the standardized uptake value ratio (SUVR), brain amyloid smoothing scores (BASSs), brain atrophy indices (BAIs), and their integration into shape features. Longitudinal changes and receiver operating characteristic analyses assessed the predictive power of these biomarkers. Results: Among 180 patients with MCI, 76 (42.2%) were converters, who exhibited significantly higher baseline and 2-year follow-up values for SUVR, BASS, BAI, and shape features than nonconverters (*p* < 0.001). Shape features demonstrated the highest predictive accuracy for conversion, with areas under the curve of 0.891 at baseline and 0.898 at 2 years. Percent change analyses revealed significant increases in brain atrophy; amyloid deposition changes showed a paradoxical decrease in converters. Additionally, strong associations were observed between longitudinal changes in shape features and neuropsychological test results. Conclusions: The integration of amyloid PET and MRI biomarkers enhances the prediction of AD progression in patients with MCI. These findings support the potential of combined imaging approaches for early diagnosis and targeted interventions in AD.

## 1. Introduction

Alzheimer’s disease (AD) is the most common form of dementia, characterized by progressive neurodegeneration and cognitive decline, and it affects millions of people worldwide [1]. As the global population ages, the prevalence of AD continues to rise, posing considerable challenges to healthcare systems [2]. Early detection and intervention are crucial for managing AD, as they offer the potential to slow disease progression and improve the quality of life of affected individuals [3]. Mild cognitive impairment (MCI) is often regarded as a prodromal stage of AD, representing a critical window for early diagnosis and intervention [4].

Recent studies have highlighted the dual pathology of AD, involving both amyloid plaques and tau pathology. Amyloid plaques, formed by extracellular deposits of amyloid-beta (Aβ) peptides, are an early hallmark of AD [5]. Meanwhile, tau pathology, characterized by intracellular neurofibrillary tangles (NFTs), composed of hyperphosphorylated tau proteins, has been strongly associated with neuronal loss and cognitive decline [6]. The integration of amyloid and tau PET imaging has been shown to improve the prediction of AD progression, underscoring the importance of considering both pathologies in diagnostic approaches [7].

In addition to imaging biomarkers, cerebrospinal fluid (CSF) and blood-based biomarkers have emerged as promising tools for early diagnosis. CSF biomarkers, such as Aβ42, total tau, and phosphorylated tau (p-tau181, p-tau217), provide direct evidence of AD pathology [8]. Blood-based biomarkers, which include plasma Aβ and tau proteins, offer a less invasive alternative, with potential for large-scale screening [9]. Recent studies suggest that combining plasma biomarkers with neuroimaging data can further enhance diagnostic accuracy [10].

Despite the advancements in biomarker research, current imaging techniques face limitations, such as variability in quantification, high costs, and limited accessibility. These challenges underscore the necessity of integrating multiple modalities to enhance diagnostic precision [11]. Multimodal approaches that combine PET, MRI, and CSF biomarkers have demonstrated superior predictive power over single-modality techniques [12].

Furthermore, the use of in vivo datasets, such as the Alzheimer’s Disease Neuroimaging Initiative (ADNI), and in silico datasets, which include computational models and synthetic data, has significantly advanced AD research. In silico datasets, in particular, enable the evaluation of novel biomarkers and predictive models while circumventing the ethical and logistical constraints of clinical studies [13]. Machine learning models trained on synthetic imaging data have recently demonstrated robust performance in predicting MCI-to-AD conversion [14].

To address the need for improved predictive accuracy in AD progression, this study introduces a novel approach that integrates amyloid deposition and brain atrophy data. Using a 6-year longitudinal dataset from the ADNI cohort, we investigate the temporal progression of AD biomarkers and propose shape-based composite biomarkers that integrate amyloid PET and MRI data. Our findings demonstrate that this multimodal approach achieves superior predictive performance compared to individual biomarkers. These results highlight the potential of combined imaging strategies for early diagnosis and targeted interventions in AD.

The objective of our study was to analyze the longitudinal data obtained from amyloid PET and brain MRI to identify and validate a quantitative method for predicting the risk of AD in patients with MCI. By integrating these imaging biomarkers, we aimed to enhance the accuracy of early diagnosis and provide a more reliable tool for clinical decision-making regarding the progression from MCI to AD.

## 2. Materials and Methods

### 2.1. Patients

**Figure A1:**
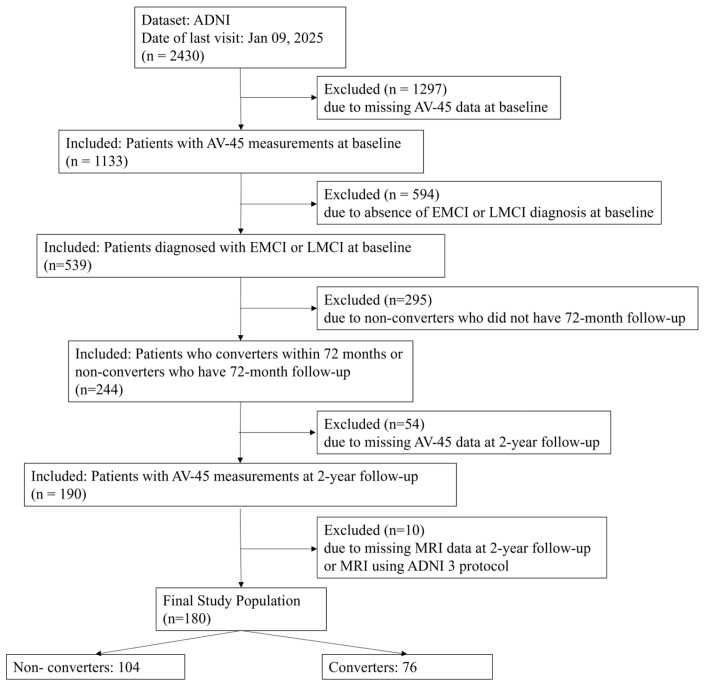
Patient selection flowchart showing the step-by-step application of inclusion and exclusion criteria to the ADNI cohort. This figure details the initial cohort size, excluded participants, and the final 180 patients included in the analysis. Abbreviations: ADNI, Alzheimer’s Disease Neuroimaging Initiative; AV-45, F-18 florbetapir.

This study used data from the Alzheimer’s Disease Neuroimaging Initiative (ADNI), a longitudinal multicenter study launched in 2004 with the primary goal of testing whether serial MRI, PET, other biological markers, and clinical and neuropsychological assessments can be combined to measure the progression of MCI and early AD. The ADNI dataset includes comprehensive clinical, imaging, genetic, and biochemical data collected from over 50 sites in the United States and Canada. For this study, we specifically selected participants diagnosed with MCI at baseline who had undergone both amyloid PET and brain MRI scans at baseline and at 2-year follow-up. The detailed patient selection process, including inclusion and exclusion criteria, is presented in Figure A1. This flowchart shows the initial ADNI cohort size, step-by-step exclusion criteria, and the final count of 180 patients included in the analysis. All data used in this analysis were sourced from the ADNI database (adni.loni.usc.edu) accessed on 9 January 2025.

In the present study, we extracted data from the ADNI 2 and GO datasets for 180 patients with MCI (early MCI or late MCI) who had baseline and 2-year follow-up AV-45 PET and brain MRI data, as well as 6-year follow-up clinical assessment data. Patients who did not undergo clinical assessments for up to 6 years and did not achieve conversion to dementia were excluded. To assess the potential for selection bias due to dropout, we conducted a comparative analysis of baseline characteristics between patients who completed the six-year follow-up and those who were excluded. As shown in Table A1, there were no significant differences between the two groups in terms of sex (*p* = 0.470), education (*p* = 0.079), APOE4 status (*p* = 0.188), or baseline Clinical Dementia Rating (CDR) scores (*p* = 0.303). Although a statistically significant difference was observed for age (*p* = 0.044), the magnitude of this difference was relatively small, indicating that it is unlikely to have introduced a substantial bias. These results indicate that the exclusion of patients owing to dropout is unlikely to have significantly affected the validity of our findings.

Patients were categorized into two groups: (i) converters, who progressed to AD within the 6-year follow-up period; and (ii) nonconverters. The onset of dementia was defined as the first time the Clinical Dementia Rating (CDR) value exceeded 1 during the follow-up period. Cognitive function was assessed using the CDR, the 13-item AD Assessment Scale–Cognitive Subscale (ADAS-cog), the Functional Activities Questionnaire (FAQ), and the Mini-Mental State Examination (MMSE).

### 2.2. AV-45 PET/CT and MRI

All PET and MRI data utilized in this study were sourced from the ADNI database and accessed at the most advanced preprocessing stage. AV-45 PET images were obtained 50–70 min post-tracer injection, followed by co-registration, temporal averaging, and standardization to a uniform voxel size of 1.5 × 1.5 × 1.5 mm. These images were collected from 57 different ADNI-participating sites.

To minimize variability arising from different PET scanners, a scanner-specific smoothing step was applied using a Gaussian smoothing kernel with a full width at half maximum (FWHM) of 6 mm. This approach was in accordance with the harmonization protocol recommended by the ADNI to ensure consistency across different imaging systems. Additionally, scanner-specific point spread function (PSF) characteristics were considered to adjust for spatial resolution differences among scanners. Notably, no additional scaling or warping was performed, thereby preserving the original brain size and shape after preprocessing.

For MRI, T1-weighted magnetization-prepared rapid gradient-echo (MPRAGE) sequences were acquired using a 1.5 T scanner, with the following parameters: TR/TE/TI = 2300/2.98/900 ms, 176 sagittal slices, within a plane FOV = 256 × 240 mm^2^, a voxel size = 1.1 × 1.1 × 1.2 mm^3^, a flip angle = 9°, and a bandwidth = 240 Hz/pix. The preprocessed imaging data used in this analysis are publicly accessible and can be downloaded from the ADNI database.

### 2.3. Quantitative PET Image Analysis

To obtain a standardized uptake value (SUV) ratio (SUVR) from the PET images, structural MRIs were initially co-registered with each participant’s AV-45 PET images. These co-registered images were used to calculate the mean weighted cortical retention uptake from the frontal, parietal, cingulate, and temporal regions. The SUVR was determined using a composite reference region that included the entire cerebellum, pons, and eroded subcortical white matter [15].

**Figure 1:**
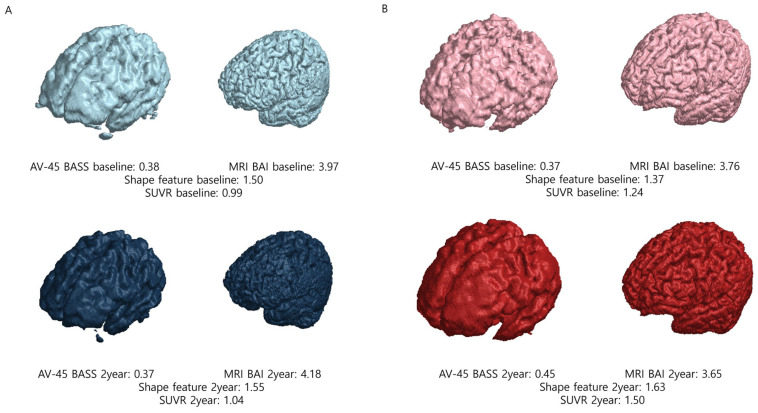
Representative examples of nonconverter and converter patients. (**A**) In nonconverter patients, the shape feature at baseline was 1.50; at the 2-year follow-up, the shape feature was 1.55, demonstrating a percent change of 0.033. (**B**) In converter patients, the shape feature at baseline was 1.37; at the 2-year follow-up, the shape feature was 1.63, demonstrating a percent change of 0.190. Abbreviations: SUVR, standardized uptake value ratio; AV-45, F-18 florbetapir; BASS, brain amyloid smoothing score; BAI, brain amyloid index.

Brain beta-amyloid deposition was quantified using a semiautomatic method, by applying the brain amyloid smoothing score (BASS). To obtain the BASS, we first obtained the volume of interest at the SUV threshold of 50% and then calculated the surface area and volume. Brain segmentation was performed by combining the segmented gray- and white-matter images using the SPM12 software package in MATLAB 2024a (MathWorks, Cambridge, UK). This method follows the unified segmentation algorithm described in a previous study [16]. A mask image was created by summing the gray- and white-matter images and applying a 50% threshold. The “regionprops3” function in MATLAB 2024a was used to calculate the surface area and volume of the mask image, and the largest value was selected. The volume and surface area of the AV-45 images were calculated based on voxel size. The BASS calculates the spherical surface area with the volume obtained here and divides it by the surface area. [17]. A representative example is shown in Figure 1.

### 2.4. Quantitative Magnetic Resonance (MR) Image Analysis

The semiautomatic quantification of brain atrophy was performed by calculating the brain atrophic index (BAI). To obtain the BAI, we first performed segmentation into gray matter and white matter. Segmented gray- and white-matter images were generated from post-processed MRI data obtained from ADNI using the SPM12 software package in MATLAB 2024a (MathWorks, Cambridge, UK). A mask image was created by combining the gray- and white-matter images. The “regionprops3” function in MATLAB 2024a was used to calculate the surface area and volume of this mask image, selecting the largest value. The BAI is calculated by dividing the surface area of the segmented brain into spherical surface areas with the same volume as the segmented brain [17]. A representative example is shown in Figure 1.

### 2.5. Shape Features Considering Both PET and MR Images

Semiautomated quantification considering brain beta-amyloid deposition and brain atrophy resulted in a variable termed the shape feature. The shape feature is obtained by multiplying the BASS and BAI [17]. The BASS reflects the spatial distribution and burden of amyloid plaques detected by AV-45 PET, while the BAI quantifies brain atrophy based on MRI data.

The rationale for using the multiplication of these two biomarkers was to more effectively capture the combined impact of amyloid deposition and brain atrophy on disease progression. The multiplicative approach was chosen to emphasize the interaction effect between amyloid burden and neurodegeneration, under the hypothesis that the simultaneous presence of both factors is more predictive of Alzheimer’s disease progression than the presence of either factor alone. Additionally, the amplification effect of the multiplication allows the shape feature to better reflect cases where both amyloid burden and atrophy are significantly elevated. This approach is consistent with previous findings suggesting that combining multiple biomarkers improves predictive accuracy for Alzheimer’s disease conversion [17].

### 2.6. Percent Change Compared at 2-Year Follow-Up

Percent change estimates were calculated using BASSs, BAIs, shape features, and SUVRs from the baseline and 2-year follow-up scans for each participant using the following formula:

$$Percent\;change\left(\%\right)=\frac{2-year\;follow-up\;value-baseline\;value}{Baseline\;value}\times 100$$

### 2.7. Statistical Analyses

Continuous data are presented as mean ± standard deviation (SD), whereas categorical data are expressed as frequencies. Independent sample t-tests were used for the analysis of continuous data, and Pearson’s Chi-squared test was used for categorical data analysis. Receiver operating characteristic (ROC) analysis with area under the curve (AUC) measurement was performed for image parameters. The ROC curve was used to compare the SUVR, shape feature, AV-45 BASS, and MRI BAI parameters. The optimal cutoff points for differentiating converters and nonconverters were determined using Youden’s J statistic (J = Sensitivity + Specificity − 1). The maximum J value for each ROC curve was selected to identify the point that maximizes the difference between sensitivity and 1-specificity. This approach was applied to the ROC curves of SUVR, AV-45 BASS, MRI BAI, and shape features. The analysis was performed using the pROC package in R.

After calculating the percent change using the aforementioned formula, independent sample t-tests were used for analysis. Spearman’s rank correlation coefficient was used to evaluate the associations between shape features and neuropsychological test results. To control for potential false positives due to multiple comparisons, we applied FDR correction using the Benjamini–Hochberg method to *p*-values. Corrected *p*-values were calculated using the p.adjust function in R with a significance threshold of 0.05.

Statistical analyses were performed using MedCalc v12.3 and R v4.0.5 (R Foundation for Statistical Computing, Vienna, Austria). Statistical significance was set at an adjusted *p* < 0.05.

## 3. Results

### 3.1. Patient Characteristics

Among the 180 patients with MCI, 76 (42.2%) developed AD during the 72-month follow-up period. Conversion to AD was significantly associated with age, APOE4 status, and baseline diagnosis. No significant differences were observed in sex or education between the converters and nonconverters. Patient demographics are shown in Table 1.

### 3.2. Imaging Parameters

The mean baseline SUVR, AV-45 BASS, MRI BAI, and shape characteristic values in the nonconverter group were 1.11 ± 0.15, 0.37 ± 0.05, 3.78 ± 0.50, and 1.40 ± 0.21, respectively; in the converter group, the mean values were 1.38 ± 0.22, 0.44 ± 0.07, 4.25 ± 0.47, and 1.86 ± 0.29, respectively. In the 2-year follow-up study in the nonconverter group, the mean SUVR, AV-45 BASS, MRI BAI, and shape feature values were 1.13 ± 0.17, 0.38 ± 0.05, 3.84 ± 0.51, and 1.45 ± 0.21, respectively; in the converter group, the mean values were 1.40 ± 0.21, 0.43 ± 0.06, 4.47 ± 0.47, and 1.92 ± 0.27, respectively. The SUVRs, AV-45 BASSs, MRI BAIs, and shape features were significantly higher in the converter group than in the nonconverter group, both at baseline and at the 2-year follow-up. The imaging parameters of the 180 patients are summarized in Table 1.

### 3.3. Receiver Operating Characteristic Curve Analysis

**Figure 2:**
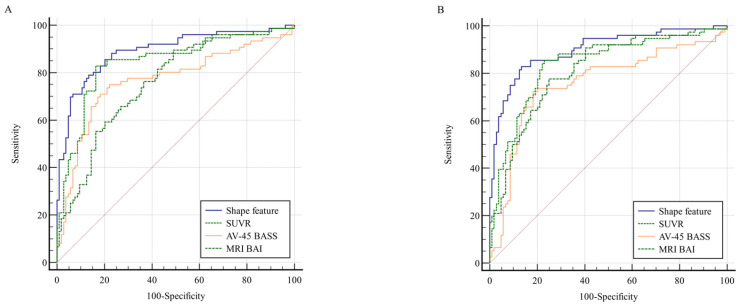
Comparison of ROC curves for shape features, SUVR, AV-45 BASS, and MRI BAI at (**A**) baseline and (**B**) 2-year follow-up. The shape feature exhibited the highest AUC value in both (**A**) and (**B**). The red diagonal line represents the chance level (random guessing) in the ROC curve. Abbreviations: ROC, receiver operating characteristic; SUVR, standardized uptake value ratio; AV-45, F-18 florbetapir; BASS, brain amyloid smoothing score; BAI, brain amyloid index; AUC, area under the curve.

The utility of the four parameters (SUVR, shape feature, AV-45 BASS, and MRI BAI) in predicting conversion to AD is demonstrated by the ROC curves (Figure 2 and Table 2). At both baseline and 2-year follow-up, the shape feature exhibited the highest AUC value. At baseline, the ROC curves of the shape feature and SUVR were not significantly different. However, at the 2-year follow-up, the shape feature was significantly different when compared to the SUVR ROC curve (*p* = 0.006). In contrast, comparisons between the shape feature and AV-45 BASS or MRI BAI revealed significant differences at both baseline and at 2-year follow-up. The results suggest that the shape feature and SUVR are optimal imaging biomarkers at baseline, whereas shape feature alone is the most effective biomarker at 2-year follow-up. Optimal cutoff points for distinguishing converters and nonconverters were determined using Youden’s J statistic (J = Sensitivity + Specificity − 1). The cutoff point for each biomarker was selected based on the maximum J value observed in ROC curves. This method was applied to all imaging biomarkers (SUVR, shape feature, AV-45 BASS, and MRI BAI) to ensure a balanced trade-off between sensitivity and specificity.

### 3.4. Percent Change Compared at 2-Year Follow-Up

The comparison of percent changes at baseline and at 2-year follow-up between the nonconverter group and the converter group is summarized in Table 3. Percent changes in the shape feature, SUVR, AV-45 BASS, and MRI BAI were 3.68 ± 6.94, 1.84 ± 5.39, 1.75 ± 5.19, and 1.89 ± 4.15, respectively, in the nonconverter group, and 3.91 ± 11.32, 1.64 ± 7.32, −1.28 ± 9.75, and 5.31 ± 5.72, respectively, in the converter group. There were no significant differences in shape features and SUVR between the nonconverter group and the converter group. However, AV-45 BASS and MRI BAI were significantly different between the two groups. MRI BAI was increased in the converter group compared to that in the nonconverter group. However, AV-45 BASS did not increase in the converter group compared to that in the nonconverter group but rather decreased.

### 3.5. Correlation of the Shape Feature with Cognitive Outcomes

**Figure 3:**
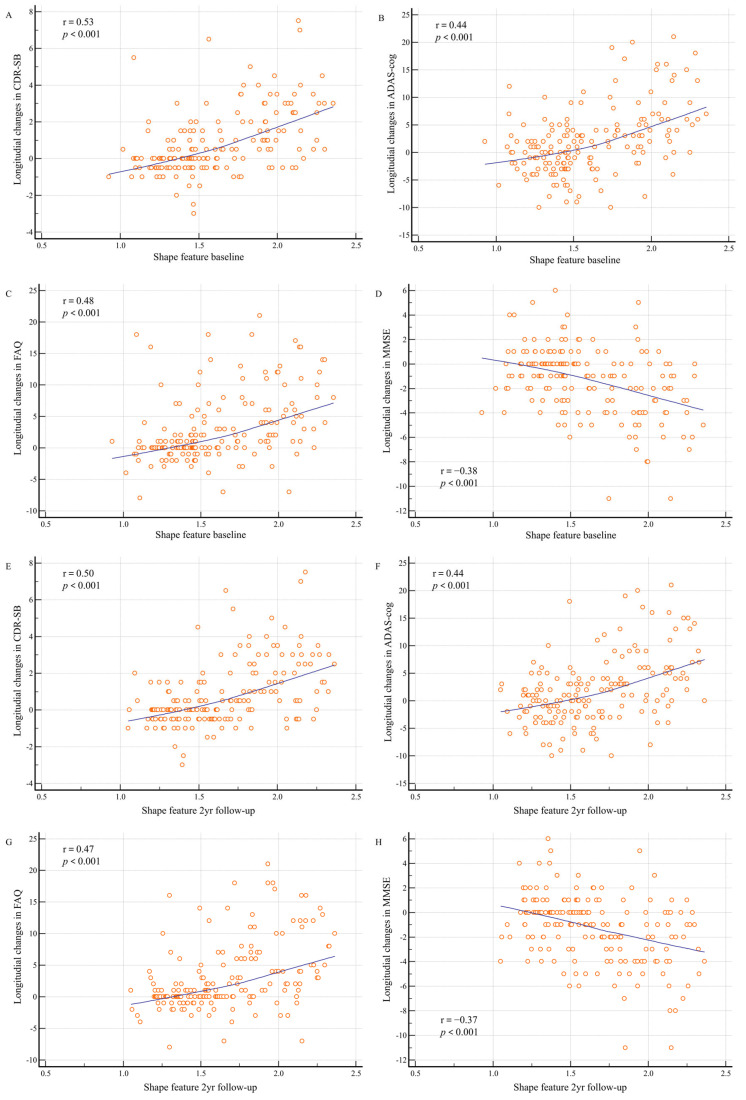
Shape feature baseline and 2-year follow-up distributions according to longitudinal changes in cognitive measurements. Changes in cognitive measurements between baseline and 2 years are shown: (**A**) between baseline shape features and Clinical Dementia Rating sum of boxes (CDR-SB); (**B**) between baseline shape features and Alzheimer’s Disease Assessment Scale–Cognitive Subscale (ADAS-cog); (**C**) between baseline shape features and Functional Activities Questionnaire (FAQ); (**D**) between baseline shape features and Mini-Mental State Examination (MMSE); (**E**) between 2-year follow-up shape features and CDR-SB; (**F**) between 2-year follow-up shape features and ADAS-cog; (**G**) between 2-year follow-up shape features and FAQ; and (**H**) between 2-year follow-up shape features and MMSE.

Baseline shape features and 2-year follow-up shape features computed from baseline PET and MR images of patients with MCI showed significant correlations with longitudinal changes in cognitive measures at 2 years (Figure 3). The baseline shape feature exhibited a positive correlation with longitudinal changes in CDR-SB (r = 0.53; *p* < 0.001), ADAS-cog (r = 0.44; *p* < 0.001), and FAQ (r = 0.48; *p* < 0.001), in addition to a negative correlation with longitudinal changes in MMSE scores (r = −0.38; *p* < 0.001). In addition, the 2-year follow-up shape feature revealed a positive correlation with the longitudinal changes in CDR-SB (r = 0.50; *p* < 0.001), ADAS-cog (r = 0.44; *p* < 0.001), and FAQ (r = 0.47; *p* < 0.001), in addition to a negative correlation with longitudinal changes in MMSE scores (r = −0.37; *p* < 0.001).

## 4. Discussion

The findings of the present study underscore the importance of integrating amyloid PET and brain MRI data to predict conversion to AD in patients with MCI. Our results demonstrate that specific imaging biomarkers, particularly those that combine amyloid deposition and brain atrophy, provide significant insights into disease progression, which is critical for early diagnosis and intervention.

Numerous recent studies have focused on predicting the conversion from MCI to AD, as well as addressing the various physiological and imaging changes that accompany this process [18,19]. One study analyzed various models for predicting this conversion and concluded that while the prediction performance was strong, further improvements could be achieved by integrating multiple factors—MRI, genetic information (such as APOE4 allele), and cognitive assessments—rather than a single determinant [18]. Additional research has demonstrated that combining multiple neuroimaging techniques, such as MRI and PET, increases prediction accuracy, yielding better prediction performance compared to the use of a single modality [19]. In the present study, we sought to predict the conversion from MCI to AD by integrating data from amyloid PET and brain MRI. In addition, several analytical methods and techniques have been introduced recently, including an innovative image analysis tool that employs AI-based quantification techniques for amyloid brain PET [20]. This area of research continues to captivate many investigators exploring the transition from MCI to AD.

Our findings suggest that the integration of amyloid PET and MRI biomarkers significantly enhances the ability to predict the progression of MCI to Alzheimer’s disease (AD). This observation is consistent with the findings of previous studies that have demonstrated the utility of multimodal imaging approaches in identifying early neurodegenerative changes associated with AD [8]. In particular, the shape feature, which combines measures of beta-amyloid deposition and brain atrophy, showed the highest predictive accuracy, reinforcing the hypothesis that a composite biomarker can capture the multifactorial nature of AD pathology more effectively than single-modality biomarkers.

However, it is also important to consider conflicting evidence. For instance, Du et al. found that plasma phosphorylated tau (p-tau217) exhibited higher discriminative accuracy for AD than amyloid PET, indicating that tau pathology may serve as a more reliable predictor of cognitive decline [21]. This highlights the need to incorporate tau PET or fluid biomarkers in future studies to validate the robustness of our composite biomarker. Moreover, Ruan et al. reported that amyloid PET abnormalities are prevalent across the AD clinical spectrum, potentially limiting its predictive value in the later stages of MCI [22]. This discrepancy emphasizes the importance of considering multiple biomarkers to capture the complex pathology of AD more accurately.

Longitudinal studies using MRI and PET play an important role in AD research. However, many previous studies have primarily focused on MRI, and although amyloid PET is recognized as an important biomarker measurement tool in AD research, it has been underutilized in longitudinal studies [23]. In the present study, the rate of change in shape features did not differ significantly between the converter and nonconverter groups. As expected, the MRI BAI, which reflects brain atrophy, demonstrated a higher rate of change in the converter group compared to in the nonconverter group. Conversely, the AV-45 BASS, an indicator of amyloid deposition, exhibited a lower rate of change in the converter group than in the nonconverter group, contrary to expectations, with a notable decrease observed in the converter group. These findings suggest that brain atrophy exerts a more substantial impact on patients with MCI as they progress to AD. This is consistent with the findings of previous studies reporting a strong correlation between the structural MRI measurements of atrophy and clinical impairments in the MCI stage [24]. Moreover, the atrophy rate observed in MRI is significantly higher in AD patients compared to in cognitively normal elderly individuals [25]. The decrease in AV-45 BASS values may be attributed to structural brain atrophy accelerating changes in amyloid deposition patterns, rather than reflecting a true slowing of amyloid accumulation. The lack of a significant difference in shape features between the two groups may be explained by the opposing effects observed in MRI BAI and AV-45 BASS. This observation is consistent with previously established temporal changes in AD biomarkers and their relationship with clinical disease progression.

Imaging biomarkers that combine measures of amyloid deposition and brain atrophy may serve as excellent predictors of conversion to AD in patients with MCI. The integration of these imaging biomarkers could have substantial implications for clinical practice [26]. The early identification of high-risk individuals allows for timely intervention, which could potentially delay the onset of AD symptoms and improve patient outcomes. Moreover, the use of these biomarkers in clinical trials could enhance the selection of participants who are most likely to benefit from therapeutic interventions, thereby improving trial efficiency. However, the longitudinal application of the shape feature imaging biomarker used in the present study appears to have limitations. In MCI patients, brain atrophy seems to exert a progressively significant effect over time, potentially overshadowing changes in amyloid deposition. Therefore, it is essential to develop and evaluate more robust biomarkers that can complement existing measures and more accurately capture amyloid deposition dynamics in the context of brain atrophy.

The association between shape features and neuropsychological test outcomes may yield valuable insights into the timing and extent of beta-amyloid deposition and brain atrophy, which are closely linked to the clinical manifestations of AD. One study indicated that increased beta-amyloid distribution and decreased gray-matter volume were associated with deficits in memory and executive function among patients with MCI [27]. Additional investigations have reported declines in both verbal and visual memory, as well as overall mental status, among individuals exhibiting beta-amyloid pathology or neurodegeneration [28]. We observed strong associations between shape features and longitudinal changes in neuropsychological test outcomes. These findings suggest the potential utility of AV-45 PET and brain MRI as indicators of neuropsychological status.

Evidence from animal models indicates that in AD, amyloid beta (Aβ) accumulates unevenly in different areas of the cortex. In a mouse model of familial AD, we demonstrated that amyloid accumulation in specific cortical areas, along with changes in cortical circuitry, differentially affect sensory, motor, and cognitive processing [29]. Other studies in different mouse models have also shown that Aβ accumulates primarily in layer 5 and the subiculum of the cortex, and over time, this accumulation spreads to other cortical areas, including CA1/2, CA3, and the dentate gyrus [30]. Human studies have shown that Aβ accumulation begins in the temporal and prefrontal cortices during the early stages of AD. Notably, individuals exhibiting rapid amyloid accumulation in the temporal and motor areas tend to have faster cognitive decline. This accumulation is closely related to cognitive decline and is considered an important factor in the early diagnosis and treatment of AD [31]. Our study seeks to enhance the understanding of AD by examining the overall distribution characteristics of Aβ accumulation rather than focusing on specific regions. We believe that there is a pressing need to develop biomarkers that can provide insights into the extent of amyloid intake occurring in specific areas of the brain. It also seems possible to utilize biomarkers that combine functional and structural imaging for other dementia diseases, including additional diseases, although further research in this area is warranted.

Despite these promising results, our study had several limitations. First, although the sample size was adequate for this analysis, it may not fully represent the broader population. Second, reliance on data from the ADNI cohort may have caused selection bias. Third, as this was an observational study rather than a rigorously controlled randomized clinical trial, our findings should be interpreted with caution. Fourth, we focused exclusively on AV-45 among several amyloid tracers, and additional studies should incorporate other amyloid tracers for a more comprehensive understanding. Future studies should aim to validate these findings in larger and more diverse populations and explore the integration of other biomarkers, such as tau PET imaging, to further refine predictive models.

Taken together, our results underscore the potential of a multimodal imaging approach for the early and accurate diagnosis of AD. However, further studies incorporating additional biomarkers such as tau PET and fluid-based markers are warranted to address the limitations highlighted by conflicting findings. By expanding the range of biomarkers, future research could refine predictive models and enhance the reliability of early diagnostic strategies for AD.

## 5. Conclusions

Our study provides strong evidence that the integration of amyloid PET and MRI data into composite biomarkers can effectively predict conversion to AD in patients with MCI. Through longitudinal analysis, we observed that brain atrophy impacts patients with MCI significantly over time. Additionally, strong associations were observed between longitudinal changes in shape features and neuropsychological test results. These findings highlight the potential for these biomarkers to play a key role in the early detection and management of AD.

## Figures and Tables

**Table 1:** Study population demographic characteristics.

Characteristics	Nonconverters	Converters	*p*-Value	FDR-Corrected *p*-Value
Number	104	76		
Age (years)	68.7 ± 6.8	73.1 ± 6.5	<0.001	0.001
Sex			0.933	0.933
Male	57 (54.8)	43 (56.6)		
Female	47 (45.2)	33 (43.4)		
Education (years)	16.8 ± 2.4	16.1 ± 2.6	0.05	0.055
APOE4			<0.001	0.001
0	65 (62.5)	24 (31.6)		
1	29 (27.9)	39 (51.3)		
2	10 (9.6)	13 (17.1)		
Diagnosis baseline			<0.001	0.001
EMCI	79 (76.0)	28 (36.8)		
LMCI	25 (24.0)	48 (63.2)		
SUVR baseline	1.11 ± 0.15	1.38 ± 0.22	<0.001	0.001
AV-45 BASS baseline	0.37 ± 0.05	0.44 ± 0.07	<0.001	0.001
MRI BAI baseline	3.78 ± 0.50	4.25 ± 0.47	<0.001	0.001
Shape feature baseline	1.40 ± 0.21	1.86 ± 0.29	<0.001	0.001
CDR baseline	0.50 ± 0.05	0.50 ± 0.00	0.394	0.414
ADAS-cog baseline	11.67 ± 4.93	20.53 ± 6.69	<0.001	0.001
FAQ baseline	0.98 ± 1.92	5.99 ± 4.76	<0.001	0.001
MMSE baseline	28.56 ± 1.51	27.17 ± 1.75	<0.001	0.001
2-year SUVR 2-year	1.13 ± 0.17	1.40 ± 0.21	<0.001	0.001
2-year AV-45 BASS	0.38 ± 0.05	0.43 ± 0.06	<0.001	0.001
2-year MRI BAI	3.84 ± 0.51	4.47 ± 0.47	<0.001	0.001
2-year shape feature	1.45 ± 0.21	1.92 ± 0.27	<0.001	0.001
2-year CDR	0.35 ± 0.29	0.72 ± 0.32	<0.001	0.001
2-year ADAS-cog	10.71 ± 5.29	25.99 ± 9.59	<0.001	0.001
2-year FAQ	1.26 ± 1.88	12.25 ± 7.50	<0.001	0.001
2-year MMSE	28.21 ± 1.76	24.53 ± 3.46	<0.001	0.001

Values are presented as means ± standard deviations or numbers (percentages). FDR-corrected *p*-values were calculated using the Benjamini–Hochberg method to control for multiple comparisons. Abbreviations: EMCI, early mild cognitive impairment; LMCI, late early mild cognitive impairment; SUVR, standardized uptake value ratio; AV-45, F-18 florbetapir; BASS, brain amyloid smoothing score; BAI, brain amyloid index; CDR, Clinical Dementia Rating; ADAS-cog, Alzheimer’s Disease Assessment Scale–Cognitive Subscale; FAQ, Functional Activities Questionnaire; MMSE, Mini–Mental State Examination.

**Table 2:** Comparison of ROC curves.

Variables	AUC	95% CI	Comparison of ROC Curves Between Each Variable and Shape Feature (*p*-Value)	Threshold	Sensitivity	Specificity	Youden’s J
Baseline							
Shape feature	0.891	0.836–0.933	-	1.60	0.79	0.87	1.65
SUVR	0.844	0.782–0.893	0.071	1.22	0.83	0.84	1.67
AV-45 BASS	0.769	0.700–0.828	<0.001	0.39	0.75	0.78	1.53
MRI BAI	0.759	0.689–0.819	<0.001	3.76	0.89	0.51	1.40
2 years							
Shape feature	0.898	0.844–0.938	-	1.67	0.83	0.87	1.69
SUVR	0.836	0.774–0.887	0.006	1.20	0.86	0.77	1.62
AV-45 BASS	0.758	0.689–0.819	<0.001	0.41	0.72	0.82	1.54
MRI BAI	0.813	0.748–0.867	0.018	4.18	0.78	0.75	1.53

Values are presented as means ± standard deviations or numbers (percentages). Abbreviations: ROC, receiver operating characteristic; AUC, area under the curve; CI, confidence interval; SUVR, standardized uptake value ratio; AV-45, F-18 florbetapir; BASS, brain amyloid smoothing score; BAI, brain amyloid index.

**Table 3:** Percent changes in selected imaging biomarkers.

Characteristics	Nonconverters	Converters	*p*-Value	FDR-Corrected *p*-Value
Number	104	76		
Shape feature percent change (%)	3.68 ± 6.94	3.91 ± 11.32	0.866	0.866
SUVR percent change (%)	1.84 ± 5.39	1.64 ± 7.32	0.833	0.866
AV-45 BASS percent change (%)	1.75 ± 5.19	−1.28 ± 9.75	0.008	0.016
MRI BAI percent change (%)	1.89 ± 4.15	5.31 ± 5.72	<0.001	0.004

Values are means ± standard deviations or numbers (percentages). FDR-corrected *p*-values were calculated using the Benjamini–Hochberg method to control for multiple comparisons. Abbreviations: SUVR, standardized uptake value ratio; AV-45, F-18 florbetapir; BASS, brain amyloid smoothing score; BAI, brain amyloid index.

## Data Availability

The ADNI dataset is available at https://adni.loni.usc.edu accessed on 9 January 2025.

## Appendix A

**Table A1:** Demographic characteristics of the study population.

Characteristics	Exclusion	Inclusion	*p*-Value
Number	295	244	
Age (years)	72.3 ± 7.8	71.0 ± 6.9	0.044
Sex			0.470
Male	159 (53.9)	140 (57.4)	
Female	136 (46.1)	104 (42.6)	
Education (years)	16.0 ± 2.6	16.4 ± 2.6	0.079
APOE4			0.188
0	155 (55.6)	117 (48.1)	
1	99 (35.5)	96 (39.5)	
2	25 (9.0)	30 (12.3)	
Diagnosis baseline			0.077
EMCI	197 (66.8)	144 (59.0)	
LMCI	98 (33.2)	100 (41.0)	
CDR baseline	0.49 ± 0.07	0.50 ± 0.05	0.303

Values are presented as means ± standard deviations or numbers (percentages). Abbreviations: EMCI, early mild cognitive impairment; LMCI, late early mild cognitive impairment; CDR, Clinical Dementia Rating.
